# Prevalence, Knowledge, Causes, and Practices of Self-Medication During the COVID-19 Pandemic in Bangladesh: A Cross-Sectional Survey

**DOI:** 10.7759/cureus.52061

**Published:** 2024-01-10

**Authors:** Sadia Mahmud Trisha, Sanjana Binte Ahmed, Md Fahim Uddin, Tahsin Tasneem Tabassum, Nur-A-Safrina Rahman, Mridul Gupta, Maisha Samiha, Shahra Tanjim Moulee, Dewan Ibna Al Sakir, Vivek Podder, Raj Kumar Agarwala, Nikita Agarwala, Priya Singhania, Suresh Kumar Tulsan

**Affiliations:** 1 Marine Biotechnology, Bangabondhu Sheikh Mujibur Rahman Maritime University, Dhaka, BGD; 2 Public Health, North South University, Dhaka, BGD; 3 Health Sciences, University of York, Heslington, GBR; 4 Health and Nutrition, Save the Children in Bangladesh, Dhaka, BGD; 5 Management Information Systems, International American University, Los Angeles, USA; 6 Clinical Services, Surjer Hashi Network, Dhaka, BGD; 7 General Medicine, Tairunnessa Memorial Medical College and Hospital, Gazipur, BGD; 8 Anesthesiology, Upazila Health Complex, Kushtia, BGD; 9 Internal Medicine, Shaheed Ziaur Rahman Medical College, Bogura, BGD; 10 Ophthalmology, Ispahani Islamia Eye Institute and Hospital, Dhaka, BGD; 11 General Surgery, Kushtia Medical College, Kushtia, BGD

**Keywords:** bangladesh, risk factors, prevalence, covid-19, self-medication

## Abstract

Introduction

During the COVID-19 pandemic, self-medication (SM) has become a critical element in the healthcare system. SM can ease the burden on hospitals and medical resources by treating minor illnesses. However, inappropriate SM practices can lead to adverse drug reactions, drug resistance, and incorrect diagnoses, resulting in poor health outcomes.

Methods

To evaluate the prevalence, knowledge, causes, and practices of SM among the Bangladeshi population during the COVID-19 outbreak, a cross-sectional survey with structured questionnaires was conducted in Chittagong City, Bangladesh, from March to May 2022. The survey included 265 participants, with an average age of 35.09 years, and a multiple-choice questionnaire was used to gather information.

Results

The study found that 64.15% of the respondents had sufficient knowledge of SM, while 35.8% had insufficient knowledge. The primary reasons for SM during the pandemic were the influence of friends/family (90.74%), fear of infection or contact with COVID-19 cases (73.15%), and fear of quarantine or self-isolation (72.22%). Analgesics/pain relievers (84%) were the most commonly used drugs for SM for COVID-19 prevention and treatment. Antiulcerants/antacid (42%), vitamin C and multivitamins (42%), and antibiotics (32%) were also frequently used.

Conclusion

This study suggests that SM is prevalent among Chittagong City residents, particularly those with less than a tertiary education. The study highlights the importance of building awareness about SM practices and taking necessary steps to control them.

## Introduction

In January 2020, the World Health Organization (WHO) declared a public health emergency in response to the emergence of COVID-19 [1]. Within six months, the global count reached approximately 20 million cases and 700,000 deaths [1]. The pandemic, marked by a fear of contracting COVID-19, limited access to healthcare, and widespread misinformation led individuals to increasingly turn to self-medication (SM). Confined at home and often relying on the Internet as their primary information source [2], many people chose SM over seeking medical attention, especially as hospitals became overcrowded.

SM refers to the practice of self-treating physical or psychological symptoms without consulting a certified physician [2]. This behavior includes reusing previously prescribed drugs, improper use of over-the-counter (OTC) medications, and purchasing drugs without prescriptions [2-3]. Globally, SM prevalence ranges from 32.5% to 81.5% [2-3]. In Bangladesh, factors, such as high treatment costs, delayed access to healthcare, dissatisfaction with health services, and a low ratio of specialists, contribute to its prevalence. While SM can alleviate the burden on hospitals and reduce treatment costs and waiting times [3-5], it also poses significant risks. These include resource wastage, pathogen resistance, antibiotic resistance [2,6], inaccurate dosages, improper administration, prolonged use, inadequate storage, drug interactions, overmedication, and the risk of addiction and abuse [7]. Such practices, driven by perceptions of symptom mildness, previous successful self-treatment, a desire for self-care, and limited medical access [6-7], are major global public health concerns.

The dangers of SM are compounded by the risks of self-diagnosis, which can lead to incorrect treatment choices and drug interactions. For instance, patients might unknowingly consume the same active ingredient under different brand names, risking double medication or harmful interactions. Incorrect administration methods, such as intravenous instead of intramuscular, are additional risks [6]. In Iran, SM has been linked to 67% of the disease burden worldwide, and its use during pregnancy is associated with 3% of congenital abnormalities [6]. In Bangladesh, common reasons for SM include high treatment costs, prior disease experiences, delayed healthcare access, and dissatisfaction with health services [6-8]. A health ministry report highlighted the scarcity of medical specialists, with only six per 10,000 individuals [7].

During the COVID-19 pandemic, the healthcare system, particularly in developing countries like Bangladesh, faced immense challenges. Periodic lockdowns and fear of contacting healthcare workers led many to avoid health facilities [7-8]. The healthcare system, already strained by a surging infection rate and limited resources, faced further challenges due to policies implemented during the pandemic. One such policy, prevalent in many countries, was the triage rule that mandated patients with COVID-19 to be treated at home unless their symptoms were severe. This not only exacerbated existing issues like the shortage of healthcare workers, insufficient hospital beds, and limited doctor availability but also led to delayed treatment for patients with worsening conditions and increased the risk of household transmission [9]. The economic impact of the nationwide lockdown also led to widespread job losses [9].

Amidst the outbreak, azithromycin and doxycycline were widely used, and a study in Dhaka City found that 77.15% of respondents used ivermectin, influenced by media reports [7]. The demand for vitamin C, multivitamins, chloroquine, and hydroxychloroquine surged, reflecting a public belief in their efficacy against COVID-19 [7,10-12]. The prevalence of SM practice varied globally: 41% in Nigeria, 45.6% in Poland, and a striking 88.33% in Bangladesh [7-10]. Such practices raise concerns about drug-induced antimicrobial resistance.

This study evaluates the prevalence and influencing factors of SM practices in Bangladesh during the COVID-19 pandemic, with the aim of guiding the development of targeted educational programs and awareness campaigns. The findings are poised to assist policymakers and health authorities in making informed decisions. While the primary focus is on prescription drugs, the study also recognizes the generally less regulated nature of OTC drugs due to their safety profile. In addition, it highlights the potential for OTC drugs to be reclassified as prescription drugs if safety concerns warrant such a change.

## Materials and methods

Study design

This cross-sectional study was conducted from March to June 2022 in Chittagong City, Bangladesh, to examine SM practices during the COVID-19 pandemic. The timeframe was strategically chosen to capture the pandemic's impact on SM practices. Participants were conveniently sampled, with the researcher visiting various city areas to recruit individuals who met these criteria and were willing to participate.

Inclusion and exclusion criteria

Included in the study were residents of Chittagong who had been ill in the past six months, aged 18 or above, and were non-medical professionals. Excluded were those unwilling to participate, who had a lack of proficiency in English, medical professionals, with a history of comorbidity, or practicing SM for over two years.

Sample size

The sample size was calculated using the formula: $n = z^2\times p\times \left ( 1-p \right )/d^2$. 

Where:

*n* is the required sample size,

*z* is the z-score corresponding to a 95% confidence level (1.96),

*p* is the estimated prevalence of SM practices (60.2% or 0.602) in Savar City, Bangladesh [6],

$(1-p)$ represents the proportion not practicing SM, and

*d *is the margin of error (5% or 0.05).

Using this formula, we initially calculated the necessary sample size to be 369 to ensure robust statistical power and reliable findings. However, the actual sample size achieved in our study was 265, which is less than the calculated requirement. This discrepancy potentially impacts the statistical power of our study, specifically the ability to detect smaller effects.

Data collection and management

Data were gathered using a structured questionnaire, divided into sections covering demographic information and detailed inquiries about SM practices, causes, and reasons (Table 1). Following data collection, we rigorously checked for accuracy and completeness before entering it into IBM SPSS Statistics for Windows, version 25 (released 2017; IBM Corp., Armonk, New York, United States) for analysis. 

**Table 1:** Study questionnaire

Sociodemographic variables of the study participants		
Questions	Options/responses	Remarks
Age		
Gender	Male/female	
Marital status	1. Single 2. Married 3. Separated 4. Divorce 5. Widow	
Do you have any Chronic disease?	1. Yes 2. No	
Educational level	1. No formal education 2. up to class 8 3. SSC or HSC 4. Graduation 5. Post-graduation	
Occupation	1. Day laborer 2. Private service 3. Govt. service 4. Unemployed 5. Housewife 6. Retired 7. Business	
Income per year	1. <20,000 per month 2. Between 20,000 and 40,000 3. Between 40,000 and 80,000 4. >60,000	
Do you have any physician in your house?	1. Yes 2. No	
Knowledge of self-medication		
Have you ever heard about self-medication?	Yes / No / I don’t know	
Can self-medication practices result in to harmful effect?	Yes / No / I don’t know	
Is self-medication for COVID-19 better than seeking medical consultation?	Yes / No / I don’t know	
Causes of self-medication		
Fear of infection or contact with suspected or known case of COVID-19	Yes / No / I don’t know	
Fear of being quarantine or self-isolation if I contract the disease	Yes / No / I don’t know	
Fear of stigma or discrimination if I contract the disease	Yes / No / I don’t know	
No drugs and treatment for COVID-19 in the health facilities	Yes / No / I don’t know	
Delay in receiving treatment at health Facilities	Yes / No / I don’t know	
Influence of friends to use self-medication to prevent COVID-19	Yes / No / I don’t know	
Influence of television, radio, newspaper & social media can lead to self-medication for COVID-19	Yes / No / I don’t know	
Practice of self-medication		
Did you self-medicate for COVID-19 in the last three months without prescription of medically qualified personnel?	Yes / No / I don’t know	
Why did you use medication(s) without prescription instead of going to a health facility?	1. Emergency illness 2. Distance to the health facility 3. Proximity of the pharmacy to home place 4. Health facility charges 5. No medicine in health facilities 6. Delaying of the hospital services 7. Did not self-medicate	
What did you use for self-medication? (can be more than one)	1. Analgesics/ antipyretics/ pain killers 2. Antiulcerants/ anti acid 3. Antihistamines 4. Antibiotics 5. Antitussives/ cough suppressants 6. Vitamins and minerals 7. Sedatives 8. Antidiarrheal 9. Herbal Products 10. No medicine	
Who prescribed the medication(s) for you?	1. Medical personnel who is unauthorized to prescribed 2. Worker in the pharmacy 3. A friend 4. myself 5. Nobody	
Where did you buy the medication	1. Pharmacy 2. Patent medicine vendor 3. Hospital 4. Faith-based outlet 5. Herbalist 6. Hawkers 7. Did not self-medicate	

Quality control and ethical considerations

To uphold the integrity of our study, rigorous quality control measures were employed. These included continuous supervision of the data collection process, conducting reliability checks on the data, and using a pre-tested questionnaire for accuracy. In terms of ethical considerations, our study was conducted with the utmost respect for participant rights. Each participant was fully informed about the study's objectives and their rights, including the freedom to withdraw at any point without any consequences. Ethical approval for this research was granted by the ethical committee of North South University (approval no. 2022/OR-NSU/IRB/02031), and all the necessary protocols involving human subjects were strictly adhered to.

Statistical analysis

The study utilized descriptive statistics (frequency, percentage, mean, and standard deviation) to summarize demographic characteristics. For exploring the associations between demographics and SM practices, we employed chi-square tests (significance level at p < 0.05). In addition, logistic regression analysis was conducted to calculate the adjusted odds ratio (AOR), thus elucidating the factors influencing SM practices. This analysis was performed using IBM SPSS Statistics for Windows, version 25, ensuring a comprehensive and accurate assessment of the data.

## Results

Characteristics of the study participants and prevalence of SM

A total of 265 respondents participated in the survey, with a mean age of 35.09 years (SD = 12.45 years). Chi-square tests were conducted to examine the association between demographic variables and SM practices. Results showed that males (n = 132) had a prevalence of 43.9% compared to females (n = 133) at 37.6% (p = 0.293). Among the age groups, respondents aged under 40 years (n = 179) had a prevalence of 40.2%, whereas those aged 40-59 years (n = 67) and over 60 years (n = 19) had higher prevalences of 50.7% and 10.5%, respectively (p = 0.006 for the age group category). Employment status, which included day laborers (n = 20, 40.0%), employed (n = 134, 47.0%), housewives (n = 40, 37.5%), and non-working individuals (n = 71, 31.0%), did not show a statistically significant association with SM practices (p = 0.160). Similarly, the level of education, categorized as zero to eight years (n = 23, 47.8%), nine to 12 years (n = 46, 41.3%), and more than 13 years (n = 196, 39.8%), was not significantly associated with SM practices (p = 0.757). Among the respondents, 64.15% (n = 170) had sufficient knowledge about SM practices, while 35.85% (n = 95) had insufficient knowledge. However, those with insufficient knowledge exhibited a significantly higher prevalence of SM practices at 81.7% (p = 0.01) (Table 2).

**Table 2:** Characteristics of the study participants and prevalence of self-medication practices.

Variables	Frequency (%)	Prevalence of self-medication	p-value
Gender			
Female	133 (50.2)	37.6	0.293
Male	132 (49.8)	43.9	
Age group			
<40	179 (67.5)	40.2	0.006
40-59	67 (25.3)	50.7	
>60	19 (7.2)	10.5	
Marital status			
Married	166 (62.6)	41.6	0.939
Single	84 (31.7)	39.3	
Widowed/divorced	15 (5.7)	40.0	
Education			
0-8 years	23 (8.7)	47.8	0.757
9-12 years	46 (17.4)	41.3	
>13 years	196 (74.0)	39.8	
Employment			
Day laborer	20 (7.5)	40.0	0.160
Employed	134 (50.6)	47.0	
Housewife	40 (15.1)	37.5	
Not-working	71 (26.8)	31.0	
Chronic disease			
No	203 (76.6)	37.9	0.09
Yes	62 (23.4)	50.0	
Any physician in house			
No	217 (81.9)	419	0.405
Yes	48 (18.1)	35.4	
Knowledge			
Sufficient	170 (64.15)	12.2	
Insufficient	95 (35.85)	81.7	

Causes for SM of COVID-19

**Figure 1:**
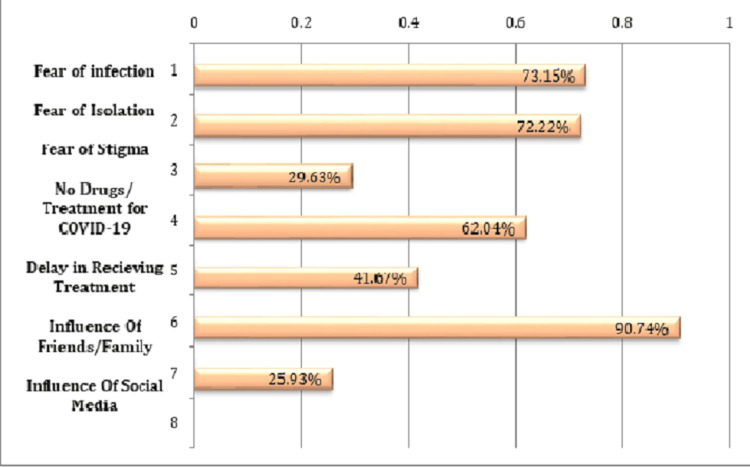
Causes of self-medication practices

A multiple-choice questionnaire was administered to determine the reasons for SM among respondents. The results are presented in Figure 1. The top reasons for SM for COVID-19 were influence from friends/family (90.74%), fear of infection or contact with a suspected or known COVID-19 case (73.15%), fear of quarantine or self-isolation (72.22%), unavailability of drugs for COVID-19 treatment in health facilities (62.04%), and delay in receiving treatment at health facilities (41.67%). Other reported reasons included influence from social media (25.93%). 

Causes for SM in the treatment and/or prevention of COVID-19

The majority of participants (66%) reported emergency illness as the primary cause of SM. Proximity to the pharmacy was the second most cited factor (20%). Other reasons included delayed access to hospital services (9%) and cost of health facilities (5%). The most commonly used drugs for SM in the treatment and prevention of COVID-19 were antipyretics/analgesics at 87%, followed by vitamin C and multivitamins at 45%, antacids at 32%, antibiotics at 30%, and herbal products at 25%. Antitussives were used by 24% of respondents, while sedatives were the least used drugs at 5%. 

Multivariate logistic regression analysis of factors associated with SM practices for COVID-19

The multivariate logistic regression model revealed that males had 1.05 times higher odds of practicing SM compared to females (OR = 1.05, 95% CI: 0.57-1.96). The odds of practicing SM among respondents aged >60 years were 92% lower compared to those below <40 years (OR: 0.08, 95% CI: 0.01-0.68). Furthermore, Table 3 shows that the respondents in the 40-59 years' age group, single, employed, housewives, not working, and those with chronic diseases had higher odds of practicing SM compared to married, day laborers, and those without chronic diseases (Table 2).

**Table 3:** Association of self-medication practice with the sociodemographic characteristics of the participants AOR: adjusted odds ratio

Variables	AOR (95% CI)
Gender	
Female	Reference
Male	1.05 (0.57–1.96)
Age group	
<40	Reference
40-59	1.41 (0.70–2.83)
>60	0.08 (0.01–0.40)
Marital status	
Married	Reference
Single	1.12 (0.51–2.47)
Widowed/divorced	0.91 (0.26–3.01)
Education	
0-8 years	Reference
9-12 years	0.31 (0.04–1.64)
>13 years	0.20 (0.03–1.02)
Employment	
Day laborer	Reference
Employed	8.67 (1.42–34.35)
Housewife	6.95 (1.09–21.60)
Not working	4.72 (0.74–22.51)
Chronic disease	
No	Reference
Yes	2.71 (1.35–5.61)
Any physician in the house	
No	Reference
Yes	0.35–1.50)

## Discussion

The study explored the knowledge, prevalence, predictors, and reasons for SM for COVID-19 prevention and treatment among individuals in Chittagong City, Bangladesh. Unlike previous studies that relied on online surveys targeting literate populations, our approach included participants from diverse sociodemographic backgrounds.

The study found that SM practices were equally common among male and female participants, predominantly in the <40 years' age group. This trend might reflect the propensity of young adults to engage in risky behavior and their lower likelihood of adhering to health recommendations. However, a higher prevalence of SM was noted in the 40-59 years' age group, aligning with findings from a study conducted in Dhaka City [7]. Our analysis also revealed a significant impact of people's knowledge, causes, and determinants on SM practices in response to COVID-19 [8]. This suggests the potential effectiveness of targeted education campaigns in enhancing adherence to SM guidelines. Notably, a higher prevalence of SM was observed among those with insufficient knowledge, echoing a similar pattern found in a Nigerian study [11]. This underscores the need for accessible, accurate information about the risks and benefits of SM, coupled with the promotion of safe, effective healthcare practices.

Our findings indicate that 40.8% of the participants engaged in SM for COVID-19 prevention and treatment. Although lower than previous studies in Dhaka City [10], this figure remains concerning due to the associated risks and adverse health outcomes. Common predictors of SM, consistent with studies in Nigeria and Dhaka City [7,11], included middle age, marital status, lower education levels, and insufficient SM knowledge. These factors suggest limited healthcare access or a perception of SM as a more convenient, cost-effective alternative. The pandemic's context has intensified these issues, potentially exacerbating health crises in unprepared regions. Addressing this challenge requires restricted media announcements, pharmacists' and drug regulators' involvement, and support from national health authorities to manage the risks associated with SM, drug shortages, and price hikes [12-14].

The study also shed light on reasons for SM, including fear of infection, stigma, influence of friends and family, treatment delays, unavailability of COVID-19 drugs, and social media influence. Emergency illness, proximity to a pharmacy, and healthcare costs were additional motivating factors. The prevalent fear and stigma surrounding COVID-19, amplified by social media and peer pressure, have led to increased SM use as people avoid hospital or clinic visits and may rely on inaccurate online information [15-17]. Notably, our findings differ from previous studies in Savar and Dhaka City, reflecting the dynamic nature of the pandemic and the evolving attitudes and behaviors of individuals toward medical care [6-7].

Most participants responded to COVID-19 prevention and treatment by using analgesics/antipyretics, such as ibuprofen, which is beneficial for alleviating COVID-19 symptoms. However, some also used acetaminophen, which, while effective in reducing fever, raises concerns about potential toxicity if used improperly. Alongside, vitamins/minerals were popular for their perceived immunity-boosting effects and easy OTC availability. Common choices also included antiulcerants, antitussives, and herbal products like echinacea and ginseng. Noteworthy is the use of antibiotics by 32% of the participants, including azithromycin. This trend, possibly influenced by social media and peers, aligns with prior research highlighting the use of azithromycin and vitamins/minerals for COVID-19 [6-7,11]. A critical concern here is the development of microbial resistance, particularly due to the unsupervised use of antibiotics. The high prevalence of antibiotic use, especially in a self-medication context, underscores the urgent need to address this issue to prevent long-term public health repercussions.

Pharmacies were identified as the primary source for purchasing medicines, attributed to their accessibility and the relatively lax regulation in the sale of medicines without prescriptions. This aligns with findings from a study in Nigeria [17]. This trend underscores a pressing need for tighter pharmacy regulations. Enhanced regulatory measures could include stringent enforcement of prescription requirements, regular audits to ensure compliance with drug dispensing guidelines, and stricter penalties for non-adherence. In addition, the implementation of educational programs for pharmacists to reinforce the importance of ethical dispensing practices is crucial. Such regulations could significantly contribute to safer medication practices, reduce the risk of drug misuse, and curb the inappropriate use of antibiotics, which is particularly pertinent in preventing the escalation of antibiotic resistance. Therefore, our findings highlight a critical area for policy intervention, suggesting that strengthening pharmacy regulations could be a pivotal step in improving public health safety, especially in the context of SM during health crises like the COVID-19 pandemic. Our study contributes valuable insights into the prevalence, predictors, and causes of SM practices for COVID-19 prevention and treatment in Dhaka City, Bangladesh. It shows the importance of enhancing knowledge about appropriate SM practices and implementing regulations to curb the unregulated sale of prescription drugs [18].

Limitations

Despite its strengths, this study had several limitations. First, information on drug dosages or treatment duration was not collected, limiting our ability to assess the effectiveness and safety of the medications used. In addition, while we gathered data on the use of traditional herbal medicines, their specific types or phytochemical compositions were not confirmed. This lack of verification of herbal remedies' details is a methodological limitation, potentially affecting the interpretation of our results, particularly concerning their efficacy, quality, and authenticity.

Moreover, the study's focus on Chittagong City limits its generalizability to other regions in Bangladesh. Another significant limitation was the relatively small sample size of our in-person surveys, which was lower than the calculated size required for optimal statistical power. This smaller sample size may have reduced the study's ability to detect true effects or associations, increasing the likelihood of type II errors. Consequently, while the study offers important insights, its findings should be considered indicative and require validation through further research with larger and more diverse sample sizes.

Furthermore, the reliance on self-reported data might have introduced a social desirability bias in reporting the prevalence of SM for purported COVID-19 prevention or treatment. Despite these limitations, the study provides valuable insights into the SM knowledge, factors, behaviors, and potential predictors among residents of Chittagong City, Bangladesh. The face-to-face interviewing approach is a notable strength of the study, enhancing the quality of the data collected.

## Conclusions

This study provides crucial insights into the prevalence, predictors, and reasons for SM among residents of Dhaka City, Bangladesh, during the COVID-19 pandemic. Our findings suggest that SM is a prevalent practice among the population, particularly for the prevention and treatment of COVID-19, with commonly used drugs, including analgesics, vitamins, antiulcerants, and antibiotics. The study identifies lack of knowledge about SM and fear of infection and isolation as significant predictors of SM. These results highlight the need for targeted awareness campaigns to counter misinformation on social media and better regulation of the pharmaceutical sector to prevent the sale of prescription drugs without proper authorization. Such interventions will not only improve the management of COVID-19 but also have broader implications for health service delivery in Bangladesh and other similar settings. Health policymakers and providers should prioritize the development of evidence-based strategies to promote responsible SM practices, ensure better access to medical advice, and enhance the quality of health care services to minimize the risk of adverse outcomes associated with SM.
